# Thermoneutrality Alters Gastrointestinal Antigen Passage Patterning and Predisposes to Oral Antigen Sensitization in Mice

**DOI:** 10.3389/fimmu.2021.636198

**Published:** 2021-03-25

**Authors:** Taeko K. Noah, Jee-Boong Lee, Christopher A. Brown, Amnah Yamani, Sunil Tomar, Varsha Ganesan, Rodney D. Newberry, Gary B. Huffnagle, Senad Divanovic, Simon P. Hogan

**Affiliations:** ^1^Department of Pathology, University of Michigan, Ann Arbor, MI, United States; ^2^Mary H Weiser Food Allergy Center, University of Michigan, Ann Arbor, MI, United States; ^3^Division of Allergy and Immunology, Cincinnati Children’s Medical Center, Cincinnati, OH, United States; ^4^Division of Pulmonary and Critical Care Medicine, Department of Internal Medicine, University of Michigan, Ann Arbor, MI, United States; ^5^Department of Medicine, Division of Gastroenterology, Washington University School of Medicine, St. Louis, MO, United States; ^6^Department of Molecular, Cellular and Developmental Biology, University of Michigan, Ann Arbor, MI, United States; ^7^Department of Pediatrics, University of Cincinnati College of Medicine, Cincinnati, OH, United States; ^8^Division of Immunobiology, Cincinnati Children’s Medical Center, Cincinnati, OH, United States; ^9^Center for Inflammation and Tolerance, Cincinnati Children’s Medical Center, Cincinnati, OH, United States

**Keywords:** food sensitization, mouse models, antigen passage, thermoneutral conditions, food allergy

## Abstract

Food allergy is an emerging epidemic, and the underlying mechanisms are not well defined partly due to the lack of robust adjuvant free experimental models of dietary antigen sensitization. As housing mice at thermoneutrality (Tn) - the temperature of metabolic homeostasis (26–30°C) – has been shown to improve modeling various human diseases involved in inflammation, we tested the impact of Tn housing on an experimental model of food sensitization. Here we demonstrate that WT BALB/c mice housed under standard temperature (18–20°C, Ts) conditions translocated the luminal antigens in the small intestine (SI) across the epithelium *via* goblet cell antigen passages (GAPs). In contrast, food allergy sensitive *Il4ra*^F709^ mice housed under standard temperature conditions translocated the luminal antigens in the SI across the epithelium *via* secretory antigen passages (SAPs). Activation of SI antigen passages and oral challenge of *Il4ra*^F709^ mice with egg allergens at standard temperature predisposed *Il4ra*^F709^ mice to develop an anaphylactic reaction. Housing *Il4ra*^F709^ mice at Tn altered systemic type 2 cytokine, IL-4, and the landscape of SI antigen passage patterning (villus and crypt involvement). Activation of SI antigen passages and oral challenge of *Il4ra*^F709^ mice with egg antigen under Tn conditions led to the robust induction of egg-specific IgE and development of food-induced mast cell activation and hypovolemic shock. Similarly, Tn housing of WT BALB/c mice altered the cellular patterning of SI antigen passage (GAPs to SAPs). Activation of SI antigen passages and the oral challenge of WT BALB/c mice with egg antigen led to systemic reactivity to egg and mast cell activation. Together these data demonstrate that Tn housing alters antigen passage cellular patterning and landscape, and concurrent oral exposure of egg antigens and SAP activation is sufficient to induce oral antigen sensitization.

## Introduction

Food allergy is an emerging epidemic (1) that is estimated to affect 32 million people in the United States (2, 3). Clinical and experimental studies have advanced our understanding of food allergy pathogenesis by revealing that food sensitization, characterized by the presence of allergen-specific IgE and CD4^+^ Th2 cells, is pathognomonic to disease (4). Although food sensitization can occur at various mucosal surfaces, including the skin and gastrointestinal tract (5, 6), the underlying mechanisms that drive food sensitization in humans have been elusive. This is in part due to mice resistance to becoming sensitized to allergens (environmental, aeroallergen, and foods) naturally, and the requirement of adjuvants such as aluminum hydroxide, cholera toxin, and staphylococcus enterotoxin B to break oral tolerance and induce sensitization to dietary antigens (7). Recent studies have demonstrated that oral exposure of food allergens in the absence of adjuvant to mice with enhanced IL4Rα signaling by a gain of function mutation (*Il4ra^F709^* mice) is sufficient to promote allergen-specific Th2 and IgE responses (8, 9). Subsequent studies revealed that heightened IL-4 signaling promoted reprogramming of regulatory T cells to Th2 (10), enhanced the number and function of ILC2 cells (11), and activated mast cells that perpetuated the IgE-mast cell response following food allergen exposure (12). Collectively, these studies revealed a role for the hematopoietic compartment in oral food sensitization in under heightened IL-4ra signaling. However, the contribution of the non-hematopoietic compartment to food sensitization and reactivity was largely unexplored (9).

Goblet cell antigen passages (GAP) are a mechanism by which the intestinal epithelium passages luminal antigens to underlying immune cells (antigen presenting cells) to mount a tolerizing response (13). The formation of GAPs in the gastrointestinal tract facilitates the tolerenergic environment by maintaining regulatory T cells, modulating dendritic cell function, and inducing IL-10 production by macrophages (13). Blockade of GAPs impairs oral tolerance as such that oral allergen exposure failed to prevent allergen specific T cell responses driven by the subcutaneous immunization (13). Accordingly, various signals including the microbiome, pathogens, epidermal growth factor (EGF), and carbachol (CCh) have been demonstrated to modulate antigen passage cellular patterning (intestinal epithelial cell population involvement) and landscape of antigen passages (villus to crypt involvement) to ensure the proper tolerizing response to the innocuous antigens (14–16). We recently demonstrated that under food allergic conditions the small intestine (SI) antigen passage cellular patterning and landscape were altered. In a food allergic state, antigen passages formed through goblet, enteroendocrine, and Paneth cells (termed SAPs) and were present in both the villus and crypt epithelium following dietary allergen exposure (17). Blockade of SAP formation inhibited a food-induced anaphylactic response suggesting the deviation in antigen passage cellular patterning and landscape is associated with allergic reactions toward dietary antigens. Currently, the contribution of altered antigen passage cellular patterning and landscape in the SI in driving food sensitization remains unexplored.

Recent investigations demonstrate that housing temperature robustly influences mice physiology (18). Vivariums in research facilities throughout the world maintain laboratory mice at 18-20°C (standard housing temperature, Ts). Under Ts conditions, mice are under chronic cold stress, exhibiting elevated stress hormones, including corticosterone (rodent stress hormone) and catecholamines (neurotransmitters to induce fight or flight responses through the sympathetic nervous system) (19, 20). Consequently, Ts housed mice exhibit elevated heart rate and oxygen consumption and dampened immune cell functions as an adaptation to the cold housing temperature (18). The ambient temperature for mice to maintain thermoneutrality is 26-30°C (Tn 30°C). At this temperature, mice do not activate thermogenic pathways to sustain core body temperature (18). Notably, at this ambient temperature Tn, the heightened stress hormone response, immune suppression, and the heightened metabolic rates observed in Ts housed mice are significantly reduced (18).

One of the limitations of studying human immunological disease processes in mice is that mice do not necessarily respond to immunological challenges similarly to humans. For example, mice are resistant to developing human disease conditions such as obesity (21), atherosclerosis (22), and severe non-alcoholic fatty liver disease (NAFLD) (23). Recent studies have revealed that the observed differences in physiological outcomes in mice are a consequence of the housing temperature at which the mice are maintained. Given the historical and recent reports of the inhibitory effects of the stress hormones on the innate and adaptive immune functions (24, 25), we examined the impact of altering the housing temperature on the intestinal immune environment and test the effects of Tn housing temperature on oral food sensitization in mice.

Herein, we show that antigen passage cellular patterning was altered in *IL4ra^F709^* mice at Ts conditions compared to WT BALB/c mice. Activation of antigen passage formation predisposed *IL4ra^F709^* mice to oral food sensitization at Ts conditions. Tn housing of *IL4ra^F709^* mice altered the antigen passage landscape and increased penetrance of food sensitization and reactivity. Tn housing of WT BALB/c mice altered the SI antigen passage cellular patterning (GAP to SAP) and landscape promoting passage formation from the villus to the crypt region. Activation of antigen passages and oral exposure of Tn conditioned housed *IL4ra^F709^* and WT BALB/c mice to egg antigen significantly exacerbated allergen-specific IgE and IgG1 responses and led to hypovolemic shock that coincided with the activation of mucosal mast cells following systemic and oral allergen challenge.

## Materials and Methods

### Animals

BALB/c WT and *Il4ra*^F709^ mice (provided from Fred D. Finkelman at Cincinnati Children’s Hospital Medical Center, CCHMC) were maintained and bred under standard housing conditions (T_S_ temperature, 18-20°C). Mice were transferred to the thermoneutral housing conditions (Tn temperature, 30-33°C) after weaning and acclimated for at least two weeks prior to experimentation. We have previously demonstrated that two weeks is sufficient to decrease serum stress hormones in WT mice (23). The Tn conditions are provided by the University of Michigan Unit for Laboratory Animal Medicine within the specific pathogen-free facility as part of IACUC-approved animal protocol. The thermostat of a small room (~ 72 ft^2^) within the facility was set at 30°C (86°F), with a 12-hour light/dark cycle. The room is equipped with a laminar flow workstation and a Rodent Cage system to maintain the mice in the room at all times. Mouse cages are maintained in a Rodent Cage system that delivers HEPA-filtered ventilated air and is equipped with an automated water supply to individual cages providing air and water at ambient temperature. The room temperature is monitored daily to ensure the temperature is maintained between 27.7-31.1 °C (82-88 °F). Six to 10-week old mice were used for all the experiments described in the study. All animals were maintained and used as approved by the Animal Care and Use Committee at CCHMC and the University of Michigan.

### Reagents

Food allergen, egg white powder from Jay Robb Enterprises (North Palm Beach, FL) was stored at -20°C until the time of use. Reagents used are as follows: Imaging antigen, lysine fixable dextran tetramethylrhodamine in 10,000 MW (Invitrogen), CCh, and paraformaldehyde (Sigma-Aldrich). Antibodies used are as follows: wheat germ agglutinin (WGA) conjugated to Alexa 488 (Invitrogen), rat anti-mouse IgE (BD Bioscience), anti rat IgG conjugated to biotin (Vector lab), avidin conjugated to HRP (BD Bioscience), or anti-mouse IgG1 (Abcam) conjugated to HRP.

### Oral Sensitization and Measurement of Food Sensitization Parameters

*Il4ra^F709^* mice were orally exposed to egg 23 mg in 400 μl water twice a week for two weeks. The mice were fasted for 5 hours and treated subcutaneously with either saline or CCh (3 μg/mouse in 100 μl saline) 15 minutes before oral food allergen exposure. The serum was harvested on day 14. On day 16, mice were challenged intravenously with 100 μg of egg allergen, and body temperature and hemoconcentration were measured as previously described (26). For oral food reactivity, *Il4ra^F709^* mice were exposed to the food allergens over 8 weeks with twice a week of oral allergen exposure following either saline or CCh treatment. Mice were orally challenged with egg allergen (23 mg) in 400 μl water every 2 weeks after 4 weeks of oral allergen exposure, and serum was harvested following each oral allergen challenge. WT BALB/c mice were exposed to egg 23 mg in 400 μl water twice a week for four weeks. Each oral allergen exposure was performed as described earlier. The serum was harvested on day 28. On day 30, mice were challenged intravenously with 100 μg of egg allergen, and blood was harvested, hemoconcentration were measured as previously described (26).

### Flow Cytometry and Single-Cell Preparation

Mononuclear cells in the lamina propria (LP) from the small intestine (SI) were isolated as previously described (27). The isolated cells were stained with anti CD3 conjugated to Brilliant Violet 605, anti CD4 conjugated to Horizon V500, anti IL-25R conjugated to allophycocyanin (APC), anti GITR conjugated to FITC, anti OX40 conjugated to Pacific Blue, anti CD8 conjugated to APC Cy7, anti c-kit conjugated to Brilliant Violet 711 and anti FcεR conjugated to phycoerythrin (PE)-Cy7. Anti γδTCR conjugated to PE, followed by counterstain with lineage markers (CD11b, CD11c, GR1, B220) conjugated with PerCP-Cy5.5. Separately the isolated cells were stained with anti B220 conjugated with APC, anti CD3 conjugated with PerCP-CY5.5, anti CD64 conjugated to Pacific Blue, anti CD11c conjugated to PE, anti CD11b conjugated to FITC, anti MHCII conjugated to APC-Cy7, and anti CD103 conjugated to PE-Cy7. Stained cells were analyzed with FACSCanto I (B.D. Bioscience) or Novocyte (ACEA Bioscience).

### RNA Extraction and Quantitative PCR Analysis

The intestinal epithelium isolated from the mononuclear cell preparation of the lamina propria were used to extract RNA as previously described (28). 1 μg of total RNA was reverse transcribed and analyzed using SYBR green based real-time PCR with the following primer sets; Il25, ACAGGGACTTGAATCGGGTC and TGGTAAAGTGGGACGGAGTTG.

### Enzyme-Linked Immunosorbent Assay (ELISA)

Serum MCPT-1 was analyzed with a kit (eBioscience) as described by the manufacture. Allergen specific IgE and IgG1 were captured with allergens coating of the plate, and the levels were detected with rat anti-mouse IgE along with biotinylated anti rat IgG or biotinylated IgG1, detection antibodies in the presence of TMB substrate solution (B.D. Bioscience). The *in vitro* cytokine capture assay (IVCCA) for IL-4 was performed as previously described (29). Serum corticosterone was measured by a kit as described by the manufacture (Arbor Assays)

### Histology, Immunofluorescence, and Microscopy

Harvested tissues were fixed and processed as previously described (17). Immunofluorescence images were acquired with a Zeiss Apotome, and bright-field images were captured with an Olympus BX51. Antigen passage was assessed and quantified per villus or crypts as previously described (17).

### Microbiome Analysis

Stools were collected from mice housed at the standard temperature. The mice were transferred to the thermoneutral temperature to collect stools after the acclimation period. DNA was isolated from the stools, as previously described (30). DNA library was prepared and sequenced with a MiSeq instrument from Illumina at the University of Michigan. Sequence data were processed and analyzed as previously described (30). Briefly, the data was processed with mothur (v.1.42.3) and put through standardization methods for community ecology using the vegan package (version 2.5.6) in R. Operational taxonomic units (OTUs) were binned at 97% similarity, and Bacteria_unclassified and any OTUs which did not cross an abundance threshold of 0.05% in any sample were excluded from the data. PERMANOVA was used for the statistical analysis.

### Statistical Analysis

Student t-test or one-way ANOVA was performed to determine statistical significance using GraphPad Prism 8 unless otherwise noted. P<0.05 is considered statistical significance unless otherwise noted.

## Results

### Antigen Passage Cellular Patterning Is Altered in *Il4ra*^F709^ Mice Under Standard Housing Conditions

**Figure 1:**
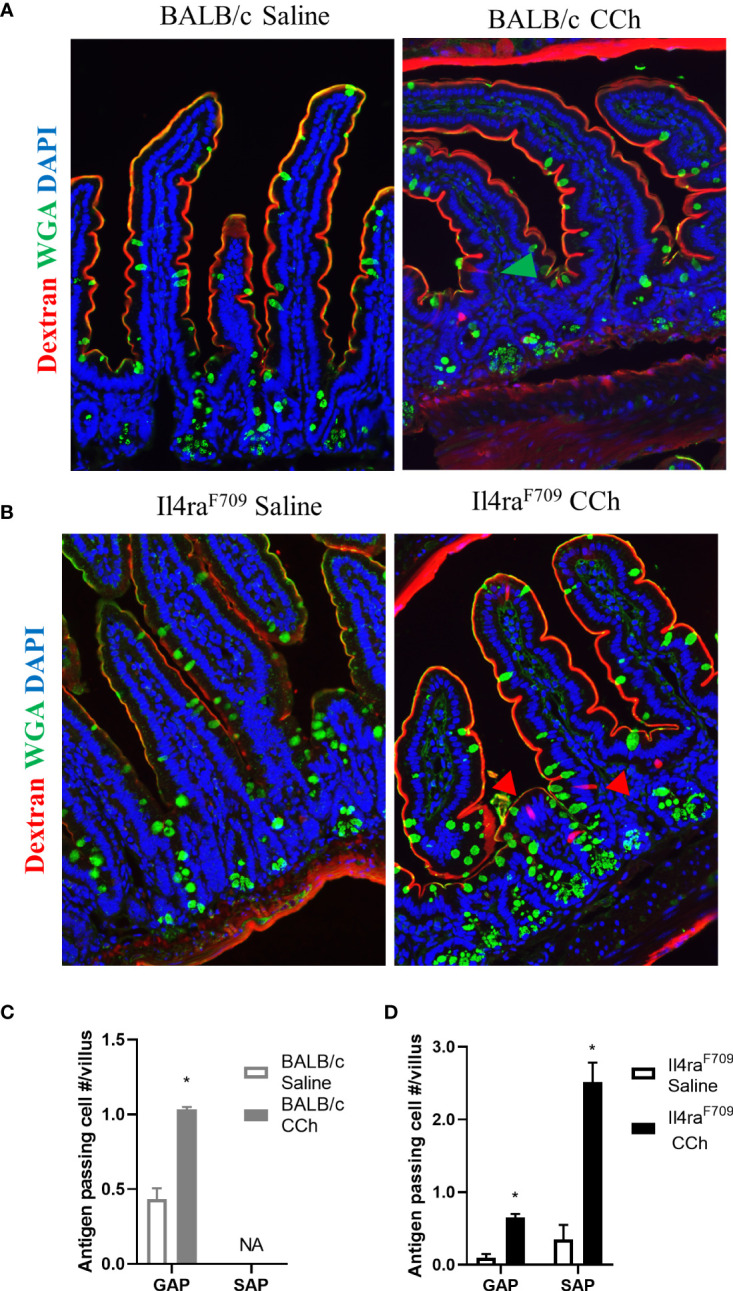
Antigen passage formation is altered in *Il4ra*^F709^. Immunofluorescence analysis for WGA (green) to identify goblet cells and dextran (red) with the small intestine of **(A)** wild type BALB/c mice and **(B)** *Il4ra^F709^* mice treated with saline or CCh. The nucleus is visualized with DAPI (blue). The green arrow points to GAP, and the red arrows point to non-GAPs. Quantification of antigen passage formation in **(C)** wild type BALB/c and **(D)** *Il4ra*^F709^ mice treated with saline or CCh. n=3 per group. *denotes statistical significance, p<0.05. NA indicates 0 value. SAP includes antigen passages across goblet and non-goblet cells.

Previous studies have reported that oral antigen exposure of *Il4ra*^F709^ and not WT BALB/C mice induce food sensitization due to the effects of heightened IL-4rα signaling on the immune compartment (31). To determine whether the differential immunological responses to dietary antigen exposure were related to differences in antigen passage formation, we evaluated steady state antigen passages in WT BALB/c and *Il4ra*^F709^ mice. We show that the frequency of antigen passages in both saline-treated *Il4ra*^F709^ and WT BALB/c mice was comparable, ~ 0.5 antigen passages per villus (**Figures 1A–D**). The antigen passages in the saline-treated WT BALB/c mice were restricted to wheat germ agglutinin (WGA)^+^ cells indicating goblet cell-restricted antigen passages (GAPs). In contrast, we observed WGA^+^ and WGA^-^ antigen passages in the saline-treated *Il4ra*^F709^ mice, indicating the presence of SAPs (**Figures 1B** and **D**). Consistent with previous reports, treatment of WT BALB/c mice with carbachol (CCh) enhanced the frequency of GAPs in the villus (**Figures 1A** and **C**, green arrow). CCh treatment of *Il4ra*^F709^ mice induced a significant increase in the frequency of WGA^+^ and WGA^-^ antigen passages (**Figures 1B, D**, red arrows). The presence of SAPs was confirmed by the demonstration that WGA^-^ antigen passages in *Il4ra*^F709^ mice were enteroendocrine cells (17) (data not shown). Collectively, these data indicate that genetic manipulation of IL4Rα is sufficient to alter SI antigen passage cellular patterning at a steady state.

To gain insight into the impact of hyperactivation of IL4-pathway on gastrointestinal immunity in *Il4ra*^F709^ mice housed under Ts conditions, we examined the intestinal immune tissues of Ts housed WT BALB/c and *Il4ra*^F709^ mice at a steady state. Flow cytometry analysis revealed that Th2, ILC2, Treg, γδ T cells, dendritic cells, and mast cell frequency in the SI were unaltered in *Il4ra*^F709^ mice (**Table 1**). However, total CD4^+^ and CD8^+^ T cells were significantly increased in the SI of *Il4ra*^F709^ mice compared to the WT BALB/c mice (**Table 1**). Consistent with previous reports, these results indicate that the intestinal immune compartment of *Il4ra*^F709^ housed at Ts condition is not biased toward Th2 at a steady state (10).

**Table 1:** List of the immune cells analyzed in the LP SI of wild type BALB/c and *Il4ra^F709^* mice.

Cell Population (absolute cell #)	WT BALB/C Ts	Y709F Ts
CD4^+^ T cells (Lin^-^ CD3^+^CD4^+^)	3.60 ± 0.27 x 10^5^	5.40 ± 0.71 x 10^5^*
CD8+ T cells (Lin^-^CD4^-^CD8^+^)	1.17 ± 0.14 x 10^5^	2.24 ± 0.41 x 10^5^*
CD4^+^ Th2 T cells (Lin^-^ CD3^+^CD4^+^ IL17RB^+^)	0.76 ± 0.11x 10^4^	1.12 ± 0.20 x 10^4^
CD4^+^ Treg cells (Lin^-^ CD3^+^CD4^+^ GITR^+^ OX40^+^)	3.87 ± 0.30 x 10^4^	4.70 ± 0.53 x 10^4^
γδ T cells (Lin^-^ CD4^-^ CD8^-^ γδ TCR^+^)	4.87 ± 0.82 x 10^4^	6.29 ± 1.1x 10^4^
ILC2 (Lin^-^ CD3^-^CD4^-^ ckit^-^ IL17RB^+^)	1.55 ± 0.17 x 10^5^	1.45 ± 0.11 x 10^5^
MC (Lin^-^CD4^-^IL17RB^-^ckit^+^FcεR^+^)	3.68 ± 0.61 x 10^4^	4.25 ± 0.73 x 10^4^
B cells (CD3^-^CD64^-^B220^+^CD11c^-^)	7.0 ± 1.1 x 10^5^	3.25 ± 1.0 x 10^5^*
Plasmatoid DCs (CD3^-^CD64^-^B220^+^CD11c^+^)	1.02 ± 0.09 x 10^5^	0.93 ± 0.09 x 10^5^
CD103+CD11b- DCs (CD3^-^CD64^-^B220^-^CD11c^+^MHCII^+^CD11b^-^CD103^+^)	1.9 ± 0.20 x 10^4^	1.7 ± 0.25 x 10^4^
CD103+CD11b+ DCs(CD3^-^CD64^-^B220^-^CD11c^+^MHCII^+^CD11b^+^CD103^+^)	5.2 ± 0.90 x 10^3^	5.7 ± 0.73 x 10^3^
CD103- DCs (CD3^-^CD64^-^B220^-^CD11c^+^MHCII^+^CD103^-^)	1.2 ± 0.09 x 10^4^	1.86 ± 0.30 x 10^4^

*denotes statistical significance. Lin, lineage; MC, mast cells; DC, dendritic cells.

### Activation of SAPs Is Associated With Increased Oral Food Sensitization Incidence in *Il4ra*^F709^ Mice Under Standard Housing Conditions

**Figure 2:**
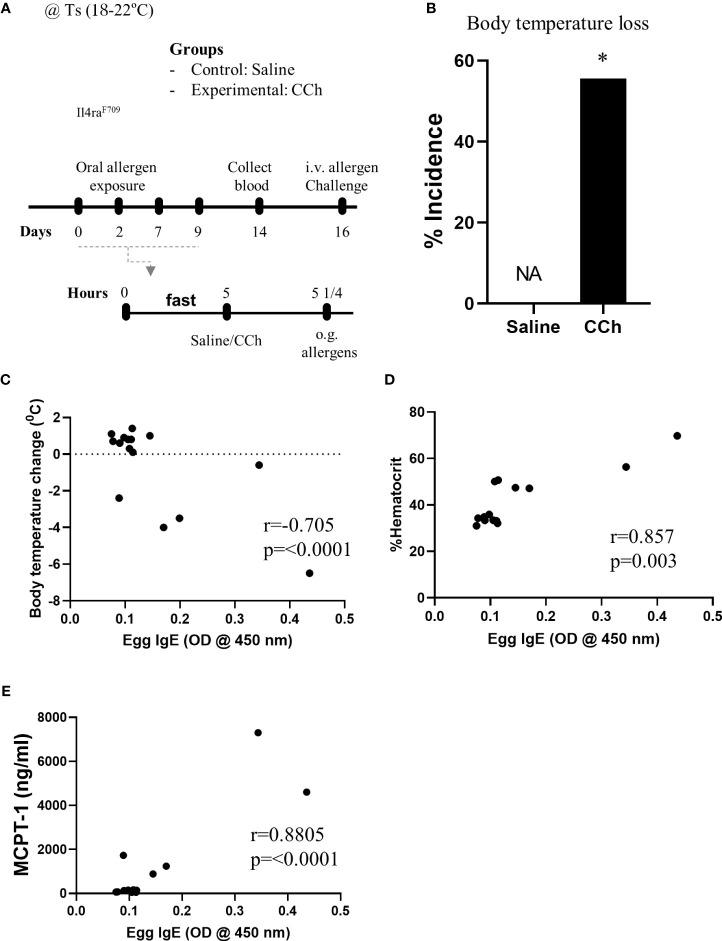
Antigen passage activation predisposes *Il4ra*^F709^ mice to food sensitization at the standard temperature. **(A)** Experimental scheme of the adjuvant free oral sensitization with Il4ra^F709^ mice. **(B)** % incidence of clinical reactivity following the systemic allergen challenge on day 16. NA indicates 0 value. Fisher’s exact test was used for the statistical analysis of the odds ratio. **(C-E)** Correlation analysis for egg IgE and **(C)** body temperature change, **(D)** % hemoconcentration, and **(E)** MCPT-1 following the systemic allergen challenge on day 16. r indicates the Pearson correlation coefficient. CCh, carbachol; i.v., intravenous; o.g., oral gavage; Ts, standard housing temperature. n=6-8 per group. * denotes statistical significance.

Given *Il4ra*^F709^ mice are susceptible to IgE-mediated food allergy (9), and we have previously demonstrated an association between SAP formation and IgE-mediated food allergic reactions, we examined the potential role of SAPs in oral food sensitization in *Il4ra*^F709^ mice. *Il4ra*^F709^ mice received repeated oral exposure of egg followed by either vehicle (saline) or CCh treatment to induce antigen passage formation (**Figure 2A**). 16 days following the first oral exposure, mice received systemic antigen challenge to assess for sensitization. Notably, systemic antigen challenge of CCh-treated *Il4ra*^F709^ mice lead to a shock response (decrease in core body temperature) in 55% of the mice compared to 0% of the saline treated *Il4ra*^F709^ mice (**Figure 2B**). The incidence of shock following systemic allergen challenge was significantly higher in the CCh-treated *Il4ra*^F709^ mice (**Figure 2B**), suggesting that oral antigen exposure and SAP activation predisposed to sensitization in *Il4ra*^F709^ mice. This was supported by the demonstration of a strong negative correlation between antigen-specific IgE and shock response (**Figure 2C**) and a positive correlation between antigen-specific IgE and hemoconcentration (evidence of hypovolemic shock) (**Figure 2D**). Importantly, we observed a positive correlation between serum MCPT-1 (mast cell activation) and antigen-specific IgE in *Il4ra*^F709^ mice demonstrating IgE-mast cell activation. Collectively, these results demonstrate that activation of SAPs in *Il4ra*^F709^ mice was associated with antigen sensitization and IgE-mast cell-mediated systemic reactions.

### Thermoneutral Housing Has Minimum Effects on the Gastrointestinal Immune Compartment in *Il4ra*^F709^ Mice

**Figure 3:**
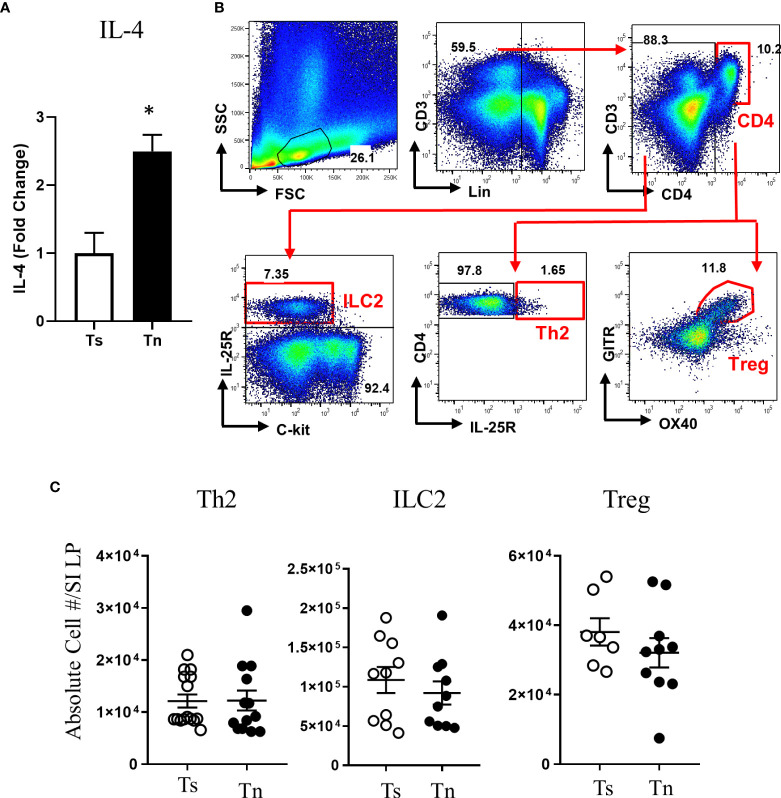
Thermoneutral housing has minimum effects on the gastrointestinal immune cells in *Il4ra*^F709^ mice. **(A)** serum IL-4 was assessed by *in vitro* cytokine capture assay (IVCCA) in Ts and Tn housed Il4ra^F709^ mice. **(B)** Gating strategies for flow cytometry analysis of the small intestinal lamina propria for ILC2, Th2 and Treg. **(C)** Flow cytometry analysis of small intestinal lamina propria for CD4+ Th2 (Lin^-^ CD3^+^CD4^+^ IL17RB^+^), ILC2 (Lin-CD4-CD8-ckit-IL17RB+), and Treg (Lin^-^ CD3^+^CD4^+^ GITR^+^ OX40^+^) from Ts and Tn housed Il4ra^F709^ mice. These immune cells were identified with the panel of markers described in **Table 1** and presented as the absolute cell number in the lamina propria (LP) of the small intestine (SI). n=5 per group for **(A)**. n=7-10 per group for **(B)**. * denotes statistical significance.

We have previously reported that Tn housing decreases serum stress hormones in WT C57BL/6 mice (23). To confirm the Tn housing effect across the different mouse strains, we examined the systemic stress hormone level in the WT BALB/c mice housed under Ts and Tn conditions. Systemic corticosterone level was significantly decreased in the Tn housed WT BALB/c mice (Ts 897.2 ± 74.2 ng/ml and Tn 125.6 ± 29.6 ng/ml serum; mean ± SEM, n = 7 – 9 mice per group; p < 0.0001), indicating that Tn housing reduces systemic corticosterone levels in different strains of mice. Examination of the effect of Tn housing on systemic Type-2 cytokine levels, revealed 1.2-fold increase in IL-4 (n = 7 - 8 p < 0.05) and 2-fold increase in IL-13 (n = 7 – 8, p < 0.01) levels in WT C57BL/6 mice. Consistent with this, serum IL-4 levels were significantly increased in Tn- vs Ts-housed *Il4ra*^F709^ mice (**Figure 3A**). Given the impact of Tn on corticosterone and IL-4 levels and their respective role in immune function (19, 32), we examined the impact of Tn housing on the intestinal immune cells within the LP SI of *Il4ra*^F709^ mice. We revealed that LP SI CD4^+^ Th2 (CD3^+^ CD4^+^ IL-17RB^+^), ILC2 (Lin^-^ CD4^-^ CD8^-^ c-kit^-^ IL17RB^+^), or the regulatory T (Tregs) (Lin^-^ CD3^+^ CD4^+^ GITR^+^ OX40^+^) cells were not significantly different between Ts- and Tn-housed *Il4ra*^F709^ mice (**Figures 3B** and **C**). Furthermore, colonic LP and mesenteric lymph node immune cells were comparable between Ts and Tn housed *Il4ra*^F709^ mice (data not shown and **Supplementary Figure 1**). Consistent with these observations, SI epithelial *Il25* mRNA levels were similar between Ts and Tn housed *Il4ra*^F709^ mice suggesting that housing temperature has very little impact on pro type 2 immune phenotype at a steady state (data not shown). Taken together, the data suggest that Tn housing did not alter the gastrointestinal immune compartment in *Il4ra*^F709^ mice.

### Thermoneutral Housing Has Minimal Effects on the Intestinal Microbiome of Il4ra^F709^ Mice

Given the previously described effect on Tn housing on the microbiome (23) and that intestinal dysbiosis has been associated with the experimental and clinical food sensitization (33–36), we analyzed the intestinal microbiome of *Il4ra*^F709^ mice housed at Ts and Tn conditions. Analysis of 16 S rRNA gene content high-throughput sequencing of V4 amplicons from fecal samples revealed no significant separation between the microbiome of Ts and Tn housed *Il4ra*^F709^ mice (**Supplementary Figure 2**, p = 0.075). To further dissect the potential impact of the housing conditions on the microbial community of *Il4ra*^F709^ mice, we performed additional analysis on the microbial sequence data. First, we identified the top 50 OTUs abundant in the *Il4ra*^F709^ mice housed at Ts conditions, then compared their % relative abundance against Tn housed *Il4ra*^F709^ mice. All but one OTUs among them, including OTU0004 (Prevotella) and OTU0008 (Bacteroides) were similarly represented between Ts and Tn housed *Il4ra*^F709^ mice supporting the principal component analysis (**Supplementary Figure 2B**). Interestingly, % relative abundance of OTU0116 (Clostridium_XIVa) was significantly reduced in the Tn housed compared to Ts housed *Il4ra*^F709^ mice (**Supplementary Figure 2B**, p = 0.046). These data indicate that Tn housing has a minimum impact on the composition of the intestinal microbiome in *Il4ra*^F709^ mice.

### The Thermoneutral Housing Altered Antigen Passage Landscape in *Il4ra*^F709^ Mice

**Figure 4:**
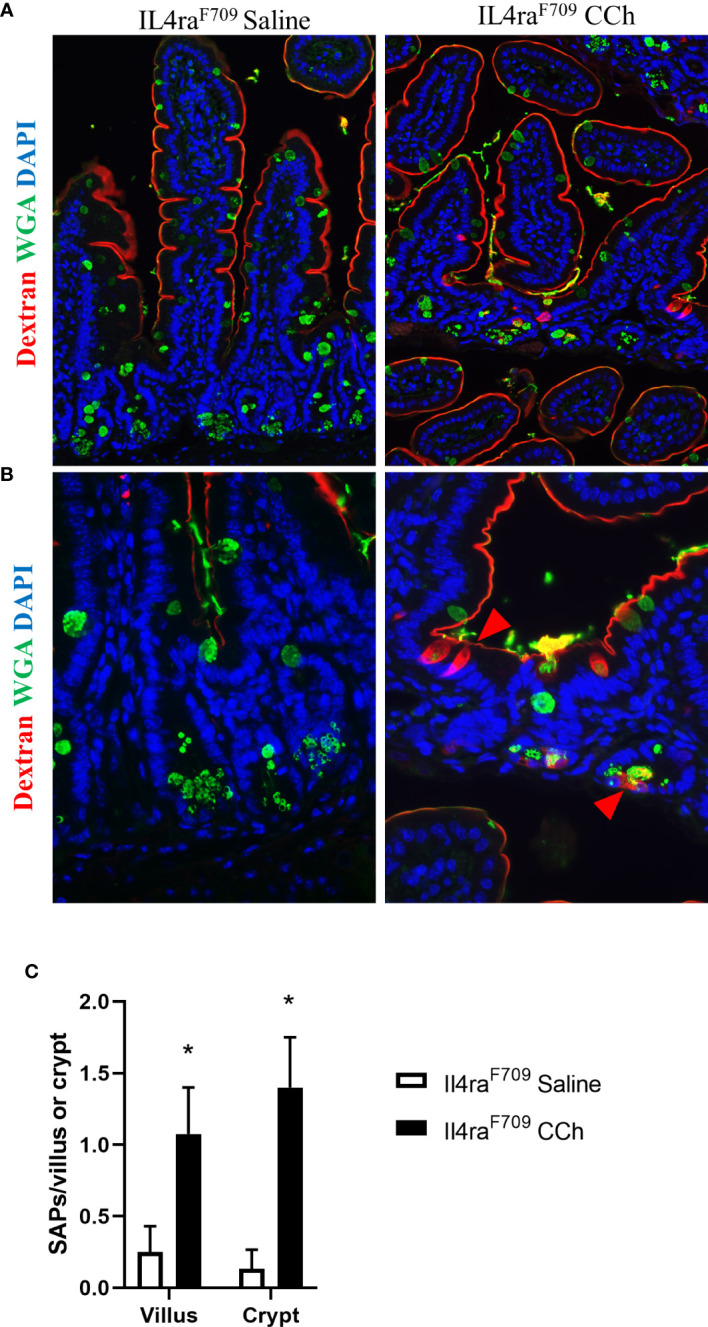
Thermoneutral housing temperature alters structural patterning of antigen passage formation in *Il4ra^F709^* mice. (A&B) Immunofluorescence analysis for WGA (green) and dextran (red) with the small intestine of Il4ra^F709^ mice treated with saline or CCh that are housed at the thermoneutral temperature. **(A)** shows a lower magnification image and **(B)** shows a higher magnification image. The nucleus is visualized with DAPI (blue). **(C)** Quantification of antigen passage formation for villi and crypts. n=2-3 per group. * denotes statistical significance.

With the observed increase in systemic IL-4 levels in Tn-housed *Il4ra*^F709^ mice and knowledge that cellular patterning of antigen passages are sensitive to IL-4rα signaling (17), we examined the impact of housing temperature on antigen passage formation in *Il4ra*^F709^ mice. Similar to the Ts housing condition, saline treated *Il4ra*^F709^ mice exhibited SAPs (~ 0.2 per villi) in the villus of the SI epithelium (**Figures 4A** and **C**). CCh stimulation lead to a significant increase in SAP frequency in the villus of *Il4ra*^F709^ mice under Tn conditions (**Figure 4C**). Notably, we also observed a significant induction of crypt SAPs (~10 fold increase) in CCh-treated *Il4ra*^F709^ mice under Tn conditions (**Figures 4B, C**). The frequency of total SAP formation across the crypt-villus unit of the Tn housed *Il4ra*^F709^ mice (**Figure 4C**), ~2.0 SAPs/villus + crypt unit) was similar to that observed in the Ts-housed *Il4ra*^F709^ mice (**Figure 1D**, ~2.0 SAPs/villus). These studies suggest that Tn conditions do not alter antigen passage frequency or cellular patterning but altered the antigen passage landscape in *Il4ra*^F709^ mice.

###  Thermoneutral Housing and SAP Activation Promotes Oral Food Reactivity to Egg Antigen in *Il4ra*^F709^ Mice

**Figure 5:**
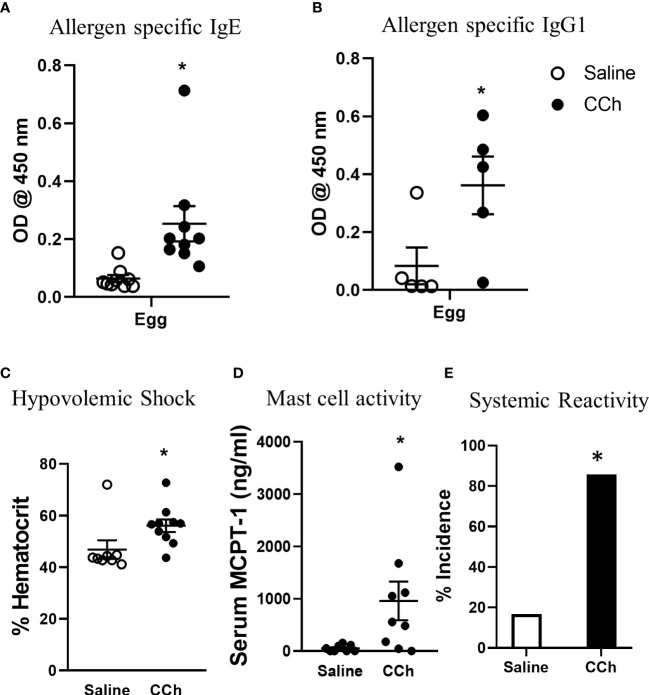
Housing *Il4ra^F709^* mice at thermoneutral temperature allow robust adjuvant free food sensitization upon activation of antigen passage formation. **(A)** allergen specific IgE and **(B)** IgG1 in the serum of saline or CCh treated Il4ra^F709^ mice housed at the thermoneutral housing temperature. The graph **(B)** shows a representative experiment and has been repeated at least 3 times. Clinical reactivity was assessed by **(C)** % hematocrit and **(D)** serum MCPT-1 level following the systemic allergen challenge. The disease penetrance **(E)** was assessed by the % incidence of clinical reactivity. Fisher’s exact test was performed for the statistical analysis. n=5-6 per group per experiment. *denotes statistical significance.

**Figure 6:**
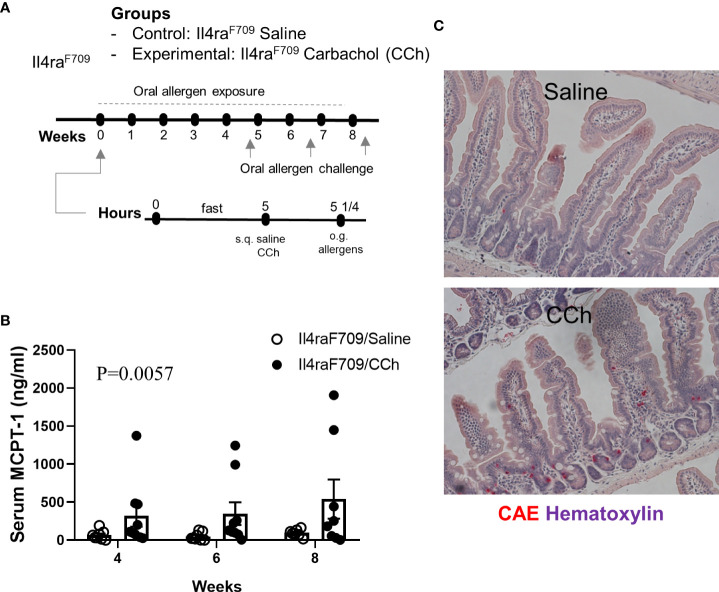
*Il4ra^F709^* mice housed at thermoneutral temperature develop food reactivity when antigen passage is activated. **(A)** experimental scheme to test the oral reactivity of Il4ra^F709^ mice at the thermoneutral housing temperature. Clinical reactivity was assessed with **(B)** the serum MCPT-1 level following oral allergen challenge. Intestinal mast cell number was examined by **(C)** CAE staining. n=8-9 per group.

To test the impact of increased systemic IL-4 levels and altered SI antigen passage landscape on oral antigen sensitization, Tn-housed *Il4ra*^F709^ mice were orally exposed to egg following either saline- or CCh-treatment (**Figure 2A**). CCh-treated *Il4ra*^F709^ mice housed under Tn conditions exhibited significantly higher levels of egg-specific IgE and IgG_1_ compared with saline-treated *Il4ra*^F709^ mice housed under Tn-conditions (**Figures 5A, B**). Systemic antigen challenge of these mice induced a shock response as evidenced by a significant increase in hemoconcentration (**Figure 5C**). Importantly, the shock response was associated with a significant increase in serum MCPT-1 (**Figure 5D**), indicating activation of mucosal mast cells. Greater than 80% of CCh-treated *Il4ra*^F709^ mice housed under Tn conditions exhibited shock (**Figure 5E**), suggesting that Tn housing increased the penetrance of oral antigen sensitization in *Il4ra*^F709^ mice (**Figures 2B** and **5E**). Longitudinal analyses (**Figure 6A**) of saline- and CCh-treated *Il4ra*^F709^ mice housed under Tn conditions revealed reactivity in CCh- and not saline-treated *Il4ra*^F709^ mice following oral antigen challenge. Moreover, CCh-treated *Il4ra*^F709^ mice housed under Tn conditions 8-weeks following sensitization began to show evidence of increased SI mast cell frequency and mucosal mast cell activation following oral antigen challenge (**Figures 6B, C**). Collectively, oral antigen exposure and SAP activation in *Il4ra*^F709^ mice housed under Tn conditions induce oral antigen sensitization and oral antigen reactivity.

### Thermoneutral Housing Alters Cellular Antigen Passage Patterning, and Activation Promotes Oral Food Reactivity to Egg Antigen in WT BALB/c Mice

**Figure 7:**
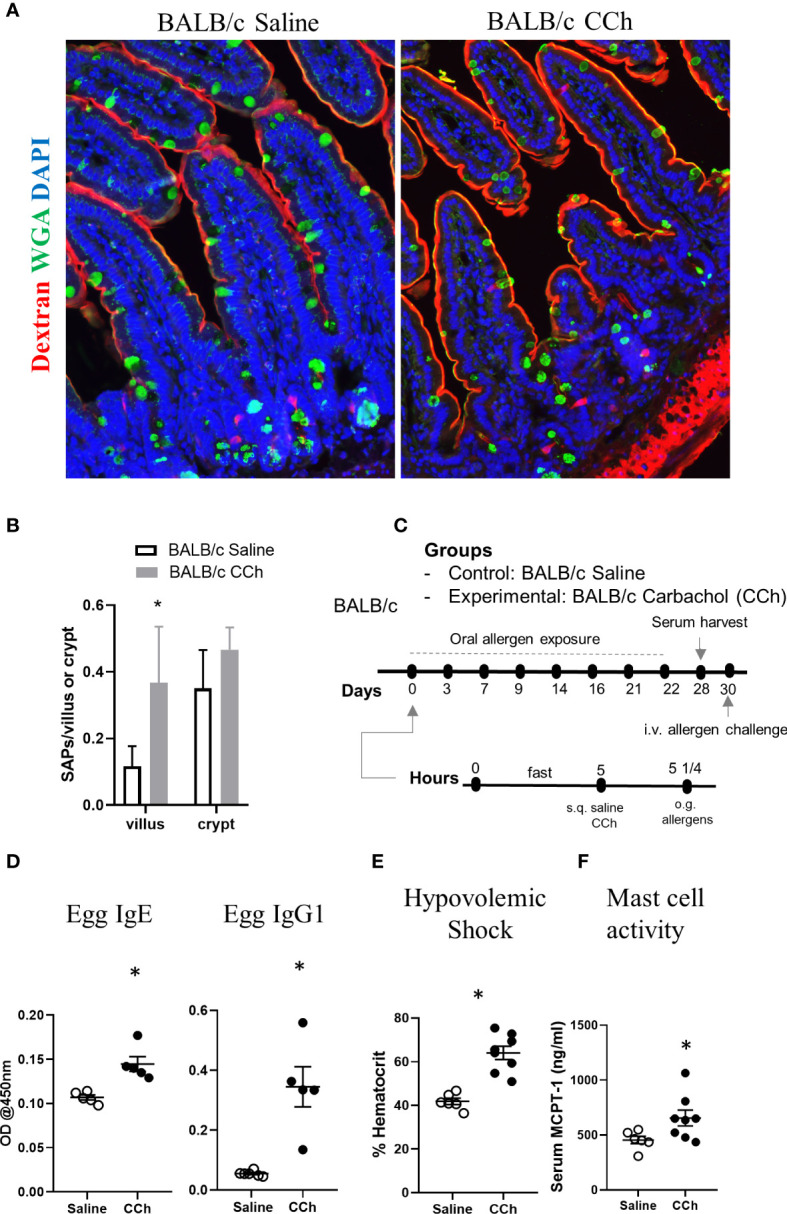
Thermoneutral housing temperature alters cellular and structural patterning of antigen passage formation and allows adjuvant free food sensitization upon activation of antigen passage formation in BALB/c mice. **(A)** Immunofluorescence analysis for WGA (green) and dextran (red) with the small intestine of BALB/c mice treated with saline or CCh that are housed at the thermoneutral temperature. **(B)** Quantification of antigen passage formation for villi and crypts. n=3 per group. **(C)** Experimental scheme of oral sensitization with BALB/c mice. **(D)** Egg specific IgE and IgG1 in the serum of saline or CCh treated BALB/c mice housed at the thermoneutral housing temperature at day 28. The graphs show representative results, and the experiment was repeated once. **(E)** % hematocrit and **(F)** serum MCPT-1 level following the systemic allergen challenge at day 30. *denotes statistical significance.

We next examined the impact of Tn conditions on antigen passage cellular patterning and oral food reactivity in WT BALB/c mice. Intriguingly, WT BALB/c mice housed under Tn conditions exhibited the presence of SI WGA^+^ and WGA^-^ antigen passages under steady state conditions suggesting that Ts to Tn conditions altered antigen passage cellular patterning and induced SAP formation (**Figures 7A, B**). Furthermore, we observed the presence of both villus and crypt antigen passages suggesting that Tn conditions also altered the antigen passage landscape (**Figures 7A, B**). To determine whether the altered SI antigen passages observed in WT BALB/c mice housed under Tn conditions predisposed to oral antigen sensitization, WT BALB/c mice under Tn conditions were orally exposed to egg allergen following either saline- or CCh-treatment and subsequently received repeated oral antigen challenge (**Figure 7C**). Repeated oral egg antigen exposure of Tn-conditioned housed WT BALB/c mice that were orally exposed to egg with CCh treatment exhibited significantly higher egg-specific IgG1 and IgE compared to saline-treated mice (**Figure 7D**). Consistent with this, repeated oral antigen challenge lead to a significant increase in hemoconcentration of CCh-treated BALB/c mice compared to the saline-treated BALB/c mice (**Figure 7E**), which coincided with the significant increase in serum MCPT-1 (**Figure 7F**) following the systemic allergen challenge. These data indicate that Tn conditions induced a shift in the antigen passage cellular patterning and landscape and that activation of antigen passage is sufficient to induce oral food sensitization and reactivity in WT BALB/c mice.

## Discussion

Herein, we demonstrate that increased IL-4 signaling is sufficient to alter cellular patterning of antigen passage in the SI. We show that activation of antigen passages in the presence of egg antigen is sufficient to promote food sensitization and reactivity. Furthermore, we show that Tn housing alters antigen cellular patterning and landscape in the SI and that activation of SAPs promotes oral antigen sensitization in the WT BALB/c mice. These studies suggest that SAPs are a mechanism by which the intestinal epithelium promotes oral food sensitization.

Goblet cell antigen passage formation and translocation of luminal antigens across GAPs have been associated with oral tolerance to microbial and dietary antigens (13, 15). In the absence of GAPs, either by the deletion of goblet cells, genetic inhibition of GAP formation, or the intraluminal administration of a GAP inhibitor, oral tolerance toward dietary antigens was disrupted, and dietary antigen specific T cell responses were observed (13). Consistent with the previous studies, our current study suggests that a shift in cellular patterning of antigen passage from GAPs to SAPs under the heightened IL-4Rα signaling conditions can promote oral sensitization. Given that Tn housing induced the alteration in cellular patterning of antigen passage in WT BALB/c mice from GAP to SAP, these results further support the notion that Tn housing promotes oral food sensitization by altering the tolerizing signals such as GAP formation. In line with this, the frequency of antigen passage formation seems to play a minimal role in oral sensitization enhanced by the Tn conditions. Similarly, the shift in landscape of antigen passage formation to the crypts seems to play a minimum role in oral food sensitization promoted by Tn housing since it was not enhanced in Tn housed BALB/c mice treated with CCh.

Type 2 cytokines IL-4 and IL-13 are known to regulate differentiation of particular intestinal epithelial cell populations, including goblet cells (37). Functionally, we previously showed that IL-13 reprogramed the cellular patterning of antigen passages formation through goblet, enteroendocrine, and Paneth cells (SAPs) in the SI (17). In the current study, antigen passage also formed through non-goblet cells of the intestinal epithelium under the heightened IL-4rα signaling condition, demonstrating the functional impact of type 2 cytokines on the intestinal epithelium. Yet, precise mechanisms by which antigen passage formation is activated by type 2 cytokines in intestinal epithelial secretory cells are still unclear. Given that intestinal epithelial of secretory cells contribute to immune responses in other pathogenic conditions such as inflammatory bowel disease (38) and infection (39), it is tempting to speculate that the type 2 cytokines may have similar effects in these disease processes. It will be important to uncover the mechanisms by which secretory antigen passages are induced by the heightened IL-4rα signaling since it will provide potential therapeutic targets to prevent induction of SAP formation.

Thermoneutral housing decreases the level of stress hormones and catecholamines which leads to a systemic increase in IL-4 levels. Stress hormones such as corticosterone are known to suppress the immune system, including ILC2 (32), decreasing type 2 cytokine production in response to cytokine activation. Also, experimental studies have recently identified intimate interactions between the sympathetic nervous system and ILC2. In these studies, it was indicated that β2 adrenergic receptor agonists suppress ILC2 mediated Th2 responses in the lung and intestine by suppressing cell proliferation and effector functions of ILC2 (40), including the production of type 2 cytokines. These studies, in aggregate, suggest that the stress hormones and catecholamines may be responsible for the systemic IL-4 increase observed in thermoneutral housing conditions. Moreover, since the catecholamines inhibit IL-4 synthesis in peripheral blood mononuclear cells *in vitro* (41), it would be important to identify the cellular source of IL-4 within peripheral blood mononuclear cells affected by catecholamines. Mast cells, basophils, and CD4^+^ T cells, which are the key immune cells involved in allergic inflammation and the cellular source of IL-4, are also under neuronal regulation (42), suggesting they may also be the cellular sources of IL-4 induced by the Tn condition.

Although it has been reported that Tn housing alters the intestinal microbiome of WT C57BL/6 mice (23), it had a minimum effect on the intestinal microbiome of *Il4ra*^F709^ mice, indicating that enhancement of food sensitization by Tn housing is independent of the intestinal microbiome. Although Clostridium_XIVa was significantly reduced in the Tn housed *Il4ra^F709^* mice, the contribution of this microbial community change in the enhancement of food sensitization driven by the thermoneutral housing condition is unclear. Since saline treated *Il4ra^F709^* mice were not sensitized at the thermoneutral conditions indicating that this change in the microbial community was not sufficient to promote sensitization. Since Clostridium_XIVa influences intestinal epithelial cell gene expression and function (35), it may contribute to enhanced food sensitization driven by Tn housing conditions through the intestinal epithelium. Thus, it would be interesting to assess the impact of Clostridium_XIVa on antigen passage formation and patterning in the future studies.

The impact of ambient housing temperature on laboratory rodents has been known for a while (43), yet the recommended housing temperature of mice has been set well below their thermoneutral temperature, likely to meet human comfort (44). As the impact of Tn housing been focused on modeling tumor immunotherapy, metabolic diseases, and infection in mice (18, 45), the current study illuminates the utility of Tn housing in modeling food allergy for the first time and offers an improved model of food allergy that does not rely on the adjuvants or genetic manipulation to drive sensitization, which more closely mimics the pathogenesis of human food allergy. As various environmental factors such as diet, microbiome, and vitamin D deficiency (46) have been proposed as risk factors for food allergy, the current experimental model provides a system to evaluate the impact of these risk factors and supports uncovering mechanisms by which these factors are driving food allergy in human.

Our current study emphasizes the relevance of the proper cellular and structural patterning of antigen passage in the homeostatic immune regulation toward dietary antigens in the SI. As a deviation of antigen passage patterning was associated with food sensitization, it would be interesting to examine the effect of risk factors associated with food allergy on antigen passage patterning to identify a mechanistic link with the development of food allergy.

## Data Availability Statement

The microbial sequence datasets for this study can be found in the NCBI BioProject as (PRJNA682183) at https://www.ncbi.nlm.nih.gov/bioproject/PRJNA682183.

## Ethics Statement

The animal study was reviewed and approved by University of Michigan and Cincinnati Children’s Medical Center.

## Author Contributions

TN, J-BL, VG, AY, and ST performed experiments. TN and SH analyzed the data, and wrote the manuscript. CB and GH processed stool samples and analyzed the microbiome. SD, RN, and GH assisted in study design and discussion. SH supervised and acquired funding. All authors contributed to the article and approved the submitted version.

## Funding

Funding was provided by National Institutes of Health grants; AI138177 (to SH), and AI112626R01(to SH), AI138348 (to GH); DK099222 (to SD); the Mary H. Weiser Food Allergy Center (MHWFAC) (to GH, SH, and TN), and the Nina and Jerry D. Luptak Endowment of the MHWFAC (to GH) and the Askwith Endowment of the MHWFAC (to SH).

## Conflict of Interest

The authors declare that the research was conducted in the absence of any commercial or financial relationships that could be construed as a potential conflict of interest.

## References

[1] Keet CA Savage JH Seopaul S Peng RD Wood RA Matsui EC. Temporal trends and racial/ethnic disparity in self-reported pediatric food allergy in the United States. Ann Allergy Asthma Immunol (2014) 112:222–9 e3. 10.1016/j.anai.2013.12.007 24428971 PMC3950907

[2] Gupta RS Warren CM Smith BM Jiang J Blumenstock JA Davis MM. Prevalence and Severity of Food Allergies Among US Adults. JAMA Netw Open (2019) 2:e185630. 10.1001/jamanetworkopen.2018.5630 30646188 PMC6324316

[3] Gupta RS Warren CM Smith BM Blumenstock JA Jiang J Davis MM. The Public Health Impact of Parent-Reported Childhood Food Allergies in the United States. Pediatrics (2018) 142(6):e20181235. Pediatrics 143 (2019). 10.1542/peds.2018-3835 30455345 PMC6317772

[4] Lopes JP Sicherer S. Food allergy: epidemiology, pathogenesis, diagnosis, prevention, and treatment. Curr Opin Immunol (2020) 66:57–64. 10.1016/j.coi.2020.03.014 32446135

[5] Samadi N Klems M Untersmayr E. The role of gastrointestinal permeability in food allergy. Ann Allergy Asthma Immunol (2018) 121:168–73. 10.1016/j.anai.2018.05.010 29803708

[6] Brough HA Nadeau KC Sindher SB Alkotob SS Chan S Bahnson HT. Epicutaneous sensitization in the development of food allergy: What is the evidence and how can this be prevented? Allergy (2020) 75:2185–205. 10.1111/all.14304 PMC7494573 32249942

[7] Schülke S Albrecht M. Mouse Models for Food Allergies: Where Do We Stand? Cells (2019) **8**(6). 10.3390/cells8060546 PMC6627293 31174293

[8] Hershey GK Friedrich MF Esswein LA Thomas ML Chatila TA. The association of atopy with a gain-of-function mutation in the alpha subunit of the interleukin-4 receptor. N Engl J Med (1997) 337:1720–5. 10.1056/NEJM199712113372403 9392697

[9] Mathias CB Hobson SA Garcia-Lloret M Lawson G Poddighe D Freyschmidt EJ. IgE-mediated systemic anaphylaxis and impaired tolerance to food antigens in mice with enhanced IL-4 receptor signaling. J Allergy Clin Immunol (2011) 127:795–805 e1-6. 10.1016/j.jaci.2010.11.009 21167580 PMC3049834

[10] Noval Rivas M Burton OT Wise P Charbonnier LM Georgiev P Oettgen HC. Regulatory T cell reprogramming toward a Th2-cell-like lineage impairs oral tolerance and promotes food allergy. Immunity (2015) 42:512–23. 10.1016/j.immuni.2015.02.004 PMC4366316 25769611

[11] Noval Rivas M Burton OT Oettgen HC Chatila T. IL-4 production by group 2 innate lymphoid cells promotes food allergy by blocking regulatory T-cell function. J Allergy Clin Immunol (2016) 138:801–11 e9. 10.1016/j.jaci.2016.02.030 27177780 PMC5014699

[12] Burton OT Noval Rivas M Zhou JS Logsdon SL Darling AR Koleoglou KJ. Immunoglobulin E signal inhibition during allergen ingestion leads to reversal of established food allergy and induction of regulatory T cells. Immunity (2014) 41:141–51. 10.1016/j.immuni.2014.05.017 PMC4123130 25017467

[13] Kulkarni DH Gustafsson JK Knoop KA McDonald KG Bidani SS Davis JE. Goblet cell associated antigen passages support the induction and maintenance of oral tolerance. Mucosal Immunol (2020) 13:271–82. 10.1038/s41385-019-0240-7 PMC7044050 31819172

[14] Kulkarni DH McDonald KG Knoop KA Gustafsson JK Kozlowski KM Hunstad DA. Goblet cell associated antigen passages are inhibited during Salmonella typhimurium infection to prevent pathogen dissemination and limit responses to dietary antigens. Mucosal Immunol (2018) 11:1103–13. 10.1038/s41385-018-0007-6 PMC6037413 29445136

[15] Knoop KA Gustafsson JK McDonald KG Kulkarni DH Coughlin PE McCrate S. Microbial antigen encounter during a preweaning interval is critical for tolerance to gut bacteria. Sci Immunol (2017) 2(18). 10.1126/sciimmunol.aao1314 PMC5759965 29246946

[16] McDonald KG Leach MR Brooke KWM Wang C Wheeler LW Hanly EK. Epithelial expression of the cytosolic retinoid chaperone cellular retinol binding protein II is essential for in vivo imprinting of local gut dendritic cells by lumenal retinoids. Am J Pathol (2012) 180:984–97. 10.1016/j.ajpath.2011.11.009 PMC3349881 22222225

[17] Noah TK Knoop KA McDonald KG Gustafsson JK Waggoner L Vanoni S. IL-13-induced intestinal secretory epithelial cell antigen passages are required for IgE-mediated food-induced anaphylaxis. J Allergy Clin Immunol (2019) 144:1058–1073 e3. 10.1016/j.jaci.2019.04.030 31175877 PMC6779525

[18] Ganeshan K Chawla A. Warming the mouse to model human diseases. Nat Rev Endocrinol (2017) 13:458–65. 10.1038/nrendo.2017.48 PMC5777302 28497813

[19] Bowers SL Bilbo SD Dhabhar FS Nelson RJ. Stressor-specific alterations in corticosterone and immune responses in mice. Brain Behav Immun (2008) 22:105–13. 10.1016/j.bbi.2007.07.012 PMC2175078 17890050

[20] Tian XY Ganeshan K Hong C Nguyen KD Qiu Y Kim J. Thermoneutral Housing Accelerates Metabolic Inflammation to Potentiate Atherosclerosis but Not Insulin Resistance. Cell Metab (2016) 23:165–78. 10.1016/j.cmet.2015.10.003 PMC4715491 26549485

[21] Osborn O Olefsky JM. The cellular and signaling networks linking the immune system and metabolism in disease. Nat Med (2012) 18:363–74. 10.1038/nm.2627 22395709

[22] Giles DA Ramkhelawon B Donelan EM Stankiewicz TE Hutchison SB Mukherjee R. Modulation of ambient temperature promotes inflammation and initiates atherosclerosis in wild type C57BL/6 mice. Mol Metab (2016) 5:1121–30. 10.1016/j.molmet.2016.09.008 PMC5081423 27818938

[23] Giles DA Moreno-Fernandez ME Stankiewicz TE Graspeuntner S Cappelletti M Wu D. Thermoneutral housing exacerbates nonalcoholic fatty liver disease in mice and allows for sex-independent disease modeling. Nat Med (2017) 23:829–38. 10.1038/nm.4346 PMC5596511 28604704

[24] Quatrini L Vivier E Ugolini S. Neuroendocrine regulation of innate lymphoid cells. Immunol Rev (2018) 286:120–36. 10.1111/imr.12707 PMC6221181 30294960

[25] Strehl C Ehlers L Gaber T Buttgereit F. Glucocorticoids-All-Rounders Tackling the Versatile Players of the Immune System. Front Immunol (2019) 10:1744. 10.3389/fimmu.2019.01744 31396235 PMC6667663

[26] Yamani A Wu D Waggoner L Noah T Koleske AJ Finkelman F. The vascular endothelial specific IL-4 receptor alpha-ABL1 kinase signaling axis regulates the severity of IgE-mediated anaphylactic reactions. J Allergy Clin Immunol (2018) 142:1159–1172 e5. 10.1016/j.jaci.2017.08.046 29157947 PMC5957775

[27] Chen CY Lee JB Liu B Ohta S Wang PY Kartashov AV. Induction of Interleukin-9-Producing Mucosal Mast Cells Promotes Susceptibility to IgE-Mediated Experimental Food Allergy. Immunity (2015) 43:788–802. 10.1016/j.immuni.2015.08.020 26410628 PMC4618257

[28] Noah TK Kazanjian A Whitsett J Shroyer NF. SAM pointed domain ETS factor (SPDEF) regulates terminal differentiation and maturation of intestinal goblet cells. Exp Cell Res (2010) 316:452–65. 10.1016/j.yexcr.2009.09.020 PMC3004755 19786015

[29] Finkelman F Morris S Orekhova T Sehy D. The in vivo cytokine capture assay for measurement of cytokine production in the mouse. Curr Protoc Immunol Chapter (2003) 6:Unit 6 28. 10.1002/0471142735.im0628s54 18432911

[30] Ashley SL Sjoding MW Popova AP Cui TX Hoostal MJ Schmidt TM. Lung and gut microbiota are altered by hyperoxia and contribute to oxygen-induced lung injury in mice. Sci Transl Med (2020) 12(556). 10.1126/scitranslmed.aau9959 PMC7732030 32801143

[31] Tachdjian R Al Khatib S Schwinglshackl A Kim HS Chen A Blasioli J. In vivo regulation of the allergic response by the IL-4 receptor alpha chain immunoreceptor tyrosine-based inhibitory motif. J Allergy Clin Immunol (2010) 125:1128–36 e8. 10.1016/j.jaci.2010.01.054 20392476 PMC2889905

[32] Yu QN Guo YB Li X Li CL Tan WP Fan XL. ILC2 frequency and activity are inhibited by glucocorticoid treatment via STAT pathway in patients with asthma. Allergy (2018) 73:1860–70. 10.1111/all.13438 PMC6175310 29542140

[33] Diesner SC Bergmayr C Pfitzner B Assmann V Krishnamurthy D Starkl P. A distinct microbiota composition is associated with protection from food allergy in an oral mouse immunization model. Clin Immunol (2016) 173:10–8. 10.1016/j.clim.2016.10.009 PMC5464391 27789346

[34] Noval Rivas M Burton OT Wise P Zhang YQ Hobson SA Garcia Lloret M. A microbiota signature associated with experimental food allergy promotes allergic sensitization and anaphylaxis. J Allergy Clin Immunol (2013) 131:201–12. 10.1016/j.jaci.2012.10.026 PMC3860814 23201093

[35] Stefka AT Feehley T Tripathi P Qiu J McCoy K Mazmanian SK. Commensal bacteria protect against food allergen sensitization. Proc Natl Acad Sci U S A (2014) 111:13145–50. 10.1073/pnas.1412008111 PMC4246970 25157157

[36] Azad MB Konya T Guttman DS Field CJ Sears MR HayGlass KT. Infant gut microbiota and food sensitization: associations in the first year of life. Clin Exp Allergy (2015) 45:632–43. 10.1111/cea.12487 25599982

[37] Andrews C McLean MH Durum SK. Cytokine Tuning of Intestinal Epithelial Function. Front Immunol (2018) 9:1270. 10.3389/fimmu.2018.01270 29922293 PMC5996247

[38] Parikh K Antanaviciute A Fawkner-Corbett D Jagielowicz M Aulicino A Lagerholm C. Colonic epithelial cell diversity in health and inflammatory bowel disease. Nature (2019) 567:49–55. 10.1038/s41586-019-0992-y 30814735

[39] Haber AL Biton M Rogel N Herbst RH Shekhar K Smillie C. A single-cell survey of the small intestinal epithelium. Nature (2017) 551:333–9. 10.1038/nature24489 PMC6022292 29144463

[40] Moriyama S Brestoff JR Flamar AL Moeller JB Klose CSN Rankin LC. beta2-adrenergic receptor-mediated negative regulation of group 2 innate lymphoid cell responses. Science (2018) 359:1056–61. 10.1126/science.aan4829 29496881

[41] Wahle M Neumann RP Moritz F Krause A Buttgereit F Baerwald CG. Beta2-adrenergic receptors mediate the differential effects of catecholamines on cytokine production of PBMC. J Interferon Cytokine Res (2005) 25:384–94. 10.1089/jir.2005.25.384 16022583

[42] Kabata H Artis D. Neuro-immune crosstalk and allergic inflammation. J Clin Invest (2019) 129:1475–82. 10.1172/JCI124609 PMC6436850 30829650

[43] Gordon CJ. Twenty-four hour rhythms of selected ambient temperature in rat and hamster. Physiol Behav (1993) 53:257–63. 10.1016/0031-9384(93)90202-Q 8446688

[44] Karp CL. Unstressing intemperate models: how cold stress undermines mouse modeling. J Exp Med (2012) 209:1069–74. 10.1084/jem.20120988 PMC3371737 22665703

[45] Hylander BL Gordon CJ Repasky EA. Manipulation of Ambient Housing Temperature To Study the Impact of Chronic Stress on Immunity and Cancer in Mice. J Immunol (2019) 202:631–6. 10.4049/jimmunol.1800621 PMC6352311 30670578

[46] Koplin JJ Allen KJ Tang MLK. Important risk factors for the development of food allergy and potential options for prevention. Expert Rev Clin Immunol (2019) 15:147–52. 10.1080/1744666X.2019.1546577 30412431

[47] Loh W Tang MLK. The Epidemiology of Food Allergy in the Global Context. Int J Environ Res Public Health (2018) 15(9). 10.3390/ijerph15092043 PMC6163515 30231558

