# Supplementary figures and images for: Thermoneutrality Alters Gastrointestinal Antigen Passage Patterning and Predisposes to Oral Antigen Sensitization in Mice
Source: Front Immunol. 2021 Mar 25;12:636198. doi: 10.3389/fimmu.2021.636198 (PMC8034294; doi:10.3389/fimmu.2021.636198)

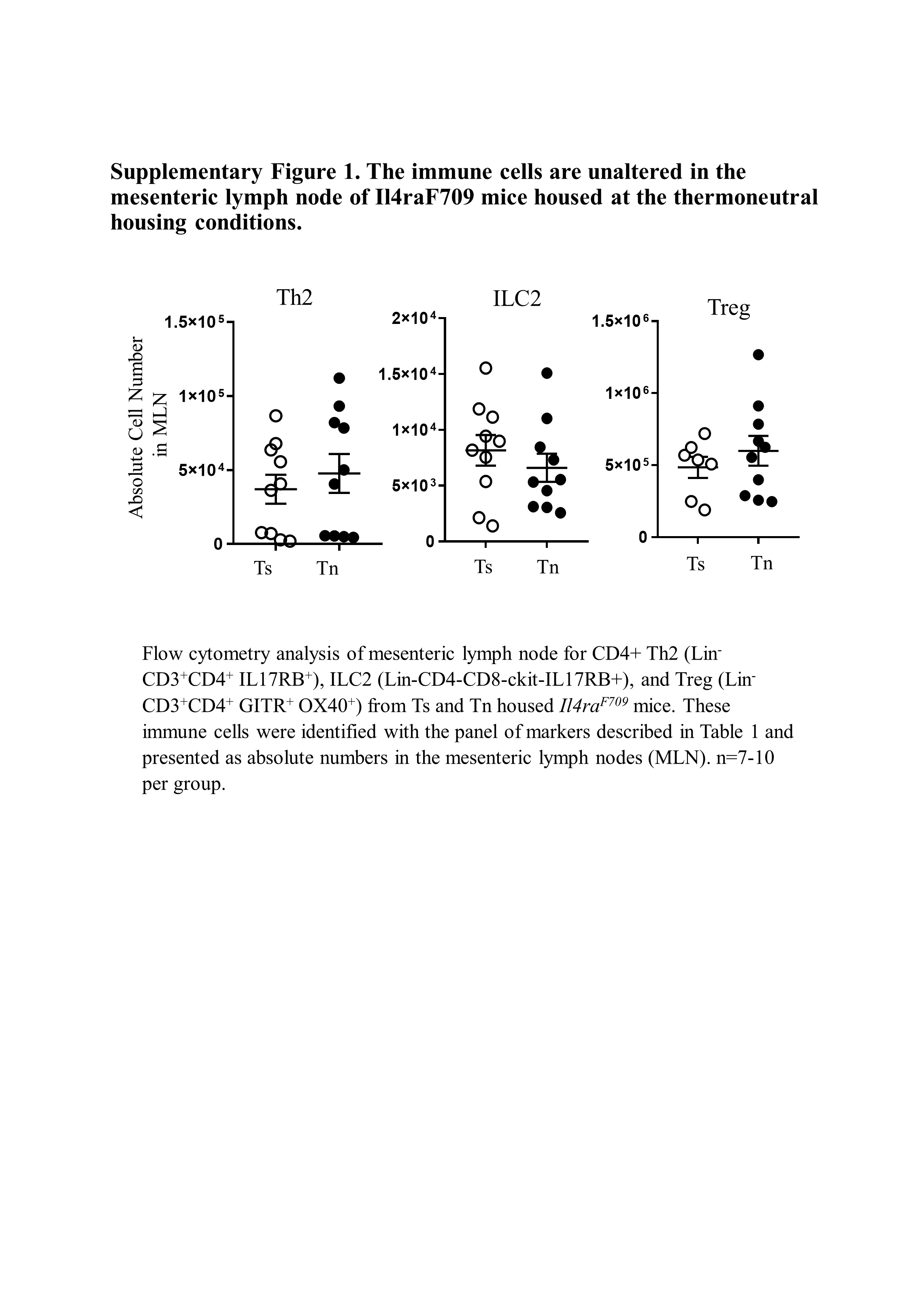

Supplement: Supplementary file 1 [file Image_1.tif]

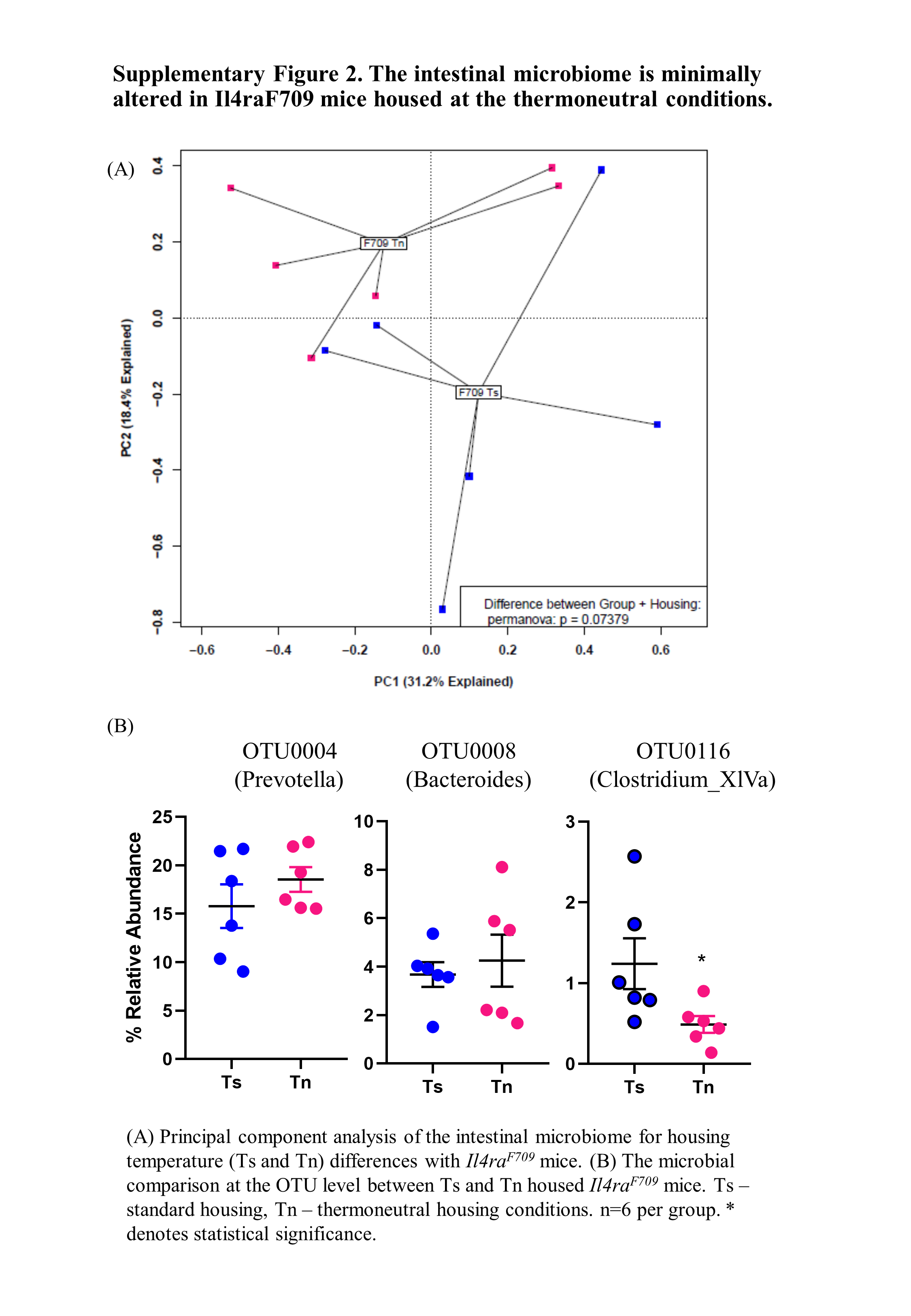

Supplement: Supplementary file 2 [file Image_2.tif]
